# A Digital Hardware Realization for Spiking Model of Cutaneous Mechanoreceptor

**DOI:** 10.3389/fnins.2018.00322

**Published:** 2018-06-08

**Authors:** Nima Salimi-Nezhad, Mahmood Amiri, Egidio Falotico, Cecilia Laschi

**Affiliations:** ^1^Medical Biology Research Center, Kermanshah University of Medical Sciences, Kermanshah, Iran; ^2^The BioRobotics Institute, Scuola Superiore Sant'Anna, Pontedera, Italy

**Keywords:** mechanoreceptor, hardware implementation, tactile sensing, spiking model, neuromorphic circuit

## Abstract

Inspired by the biology of human tactile perception, a hardware neuromorphic approach is proposed for spiking model of mechanoreceptors to encode the input force. In this way, a digital circuit is designed for a slowly adapting type I (SA-I) and fast adapting type I (FA-I) mechanoreceptors to be implemented on a low-cost digital hardware, such as field-programmable gate array (FPGA). This system computationally replicates the neural firing responses of both afferents. Then, comparative simulations are shown. The spiking models of mechanoreceptors are first simulated in MATLAB and next the digital neuromorphic circuits simulated in VIVADO are also compared to show that obtained results are in good agreement both quantitatively and qualitatively. Finally, we test the performance of the proposed digital mechanoreceptors in hardware using a prepared experimental set up. Hardware synthesis and physical realization on FPGA indicate that the digital mechanoreceptors are able to replicate essential characteristics of different firing patterns including bursting and spiking responses of the SA-I and FA-I mechanoreceptors. In addition to parallel computation, a main advantage of this method is that the mechanoreceptor digital circuits can be implemented in real-time through low-power neuromorphic hardware. This novel engineering framework is generally suitable for use in robotic and hand-prosthetic applications, so progressing the state of the art for tactile sensing.

## Introduction

Touch is a co-existing sensation required to interact with our surrounding environments (Tiwana et al., 2012; Yi and Zhang, 2017). The sensitivity provided by the sense of touch enables us to distinguish different textures and manipulate grasped objects, accurately. The sense of touch arises from receptors placed throughout the whole body and its modality is divided into three categories: cutaneous (tactile), kinesthetic and haptic (Bensmaia et al., 2008; Chaudhuri, 2011). The kinesthetic and cutaneous systems differ in terms of the location of mechanoreceptors in response to the sensory inputs. The cutaneous system relies on the receptors embedded in the skin, while the former is based on the receptors within muscles and joints. The haptic system utilizes the combined sensory inputs from both systems (Healy and Proctor, 2003; Chaudhuri, 2011). In natural contact, the mechanoreceptor cells are activated and carry information about the objects' size and shape (Kim et al., 2009; Yi et al., 2017).

The glabrous part of the human skin comprises four types of mechanoreceptors: Merkel's disks, Ruffini cylinders, Meissner and Pacinian corpuscles, each is responsible for the reception of specific stimuli and then sends tactile information by myelinated fibers to the central nervous system (CNS) for higher level perception (Pearson et al., 2011; Yi et al., 2017). These mechanoreceptors are divided into two categories, slowly adapting (SA) and fast adapting (FA). Merkel's disks are innervated by SA-I fibers and Ruffini cylinders by SA-II fibers, respond to low frequency stimuli and describe the static properties of a stimulus including skin indentations and stretch. In contrast, Meissner corpuscles are innervated by fast adapting fibers (FA-I), and Pacinian corpuscles by PC fibers (FA-II) and respond to skin vibrations (Saal et al., 2017).

Goodwin and Wheat found that humans could estimate the magnitude of the contact force and the shape and contact force information could be independently perceived by humans (Goodwin and Wheat, 1992). By applying the force stimulus to the volar surface of the index fingertip, the human ability to discriminate the 3D force stimuli was investigated (Panarese and Edin, 2011). The authors presented that the force direction was recognized mainly during the dynamic force stimulation, while the static force stimulation improved the discrimination ability only to a limited extent. Birznieks et al. (2009), reported that when forces were applied in five distinct directions, almost all the tactile afferents such as SA-I/SA-II and FA-I afferents from the whole terminal phalanx responded. The authors concluded the tactile afferents potentially contributed to the encoding of the fingertip forces.

Tactile sensing using spiking neural networks has attracted increasing attention in the recent years (Friedl et al., 2016; Oddo et al., 2016; Yi and Zhang, 2016). To discriminate local curvature of objects, Lee et al. used a fabric based binary tactile sensor array (Lee et al., 2013). The tactile signals were converted into spikes using two Izhikevich models. Lee and his collaborators (Lee et al., 2014), also applied the soft neuromorphic method for gait event detection using a low-cost, foot pressure sensor. A closed perception-action loop was constructed for classifying Braille letters (Bologna et al., 2013), by providing pressure sensor data to the leaky integrate-and-fire neurons (LIF). In contrast to Lee et al.'s work, their work was distinct in both analog-to-spike transformation model and pattern decoding algorithms (Bologna et al., 2011). By simulating the Izhikevich neuron in response to an array of four piezoresistive sensors and examining the spike timing, 10 naturalistic textures have been classified by Rongala and his coworkers (Oddo et al., 2011; Rongala et al., 2015). Finally, Oddo and his colleagues used the same sensor to transduce haptic stimulus into a spatiotemporal pattern of spikes. By delivering these spike patterns to the skin afferents of the rats through an array of stimulation electrodes, they showed a potential neuro-prosthetic approach to communicate with the rat brain (Oddo et al., 2017).

Using neural coding principles to design and implement the neuro-mimetic architectures for active perception may help in embedding neuro-prosthetic devices with sensory feedbacks. This neuromorphic implementation of touch sensing based on artificial spiking neurons can accelerate the design of new architectures for artificial tactile sensory systems for development of assistive and human rehabilitation and also industrial robotics (Kim et al., 2009; Raspopovic et al., 2014). Nevertheless, the development of a new architecture for spiking mechanoreceptor is still necessary, and hence in the present research, we directly proceed to the neuromorphic implementation in hardware. We adopt this approach utilizing the Izhikevich spiking model to convert sensor outputs to spike/burst trains conveying tactile information (Rongala et al., 2015; Oddo et al., 2016; Yi et al., 2017). This novel engineering framework is an important step toward the upcoming hardwired implementation of the mechano-neuro transduction process which is not addressed yet.

One of the most common methods to realize the neural computational models is developing hardware circuit due to its high operating efficiency for practical applications (Cassidy et al., 2011; Nazari et al., 2014a; Ranjbar and Amiri, 2016). Very large scale integration (VLSI) design can be more realistic for hardware implementations of spiking neuronal networks due to its capability to implement nonlinear models in a straightforward way (Ranjbar and Amiri, 2015; Yang et al., 2016), however the long development time and high costs of this method limit its usage (Nazari et al., 2015a,b). On the one hand, digital execution with field-programmable gate array, (FPGA) can be faster and thus FPGAs have increasing applications in the neural computing area, in recent years (Bonabi et al., 2012; Sabarad et al., 2012; Nanami and Kohno, 2016). Currently, with the advancement in HDL synthesis tools (high-level hardware description language), configurable devices (such as FPGA) can be operated as effective hardware accelerators for neuromorphic systems. Indeed, FPGA technology provides flexibility necessary for algorithm exploration while satisfying time and performance constraints (Misra and Saha, 2010; Arthur et al., 2012).

The feasibility of using FPGAs for simulation of the Izhikevich model in a pipelined manner for character recognition was explored in Rice et al. (2009). Wang et al. presented an FPGA realization of a polychronous spiking neural network for spatial-temporal patterns. The proposed network was capable of successfully recalling of spikes for the stored patterns (Wang et al., 2013). Grassia and collaborators investigated the feasibility of stochastic neuron simulation in FPGA, and realized a digital implementation for a two-dimensional neuron model (Grassia et al., 2017). In Ambroise et al. (2013a), a digital hardware implementation of a biorealistic neural network composed of 117 Izhikevich neurons which works in biological real time was described. In this way, using the Izhikevich model, a biomimetic implementation of a network of 240 CPGs (central pattern generator) in an FPGA, to implement the leech heartbeat system neural network with minimum resources was explored in Ambroise et al. (2013b). This digital system opens the way toward hybridization of biological tissue and artificial neural networks. Indeed, a hybrid interconnection between a living spinal cord and an artificial neural network to restore functional activity was demonstrated in Joucla et al. (2016). It facilitated toward the realization of a new neuroprosthesis in which an open/closed-loop bio-hybrid experiment was implemented in the neuromorphic board using uni/bi-directional communication between *in vitro* biological neuronal network and artificial neural network (Ambroise et al., 2017).

In this paper, we propose a digital neuromorphic circuit for SA-I mechanoreceptors (Merkel mechanoreceptors), and FA-I mechanoreceptors (Meissner's corpuscles) which are also important cells for surface roughness perception. First, in order to achieve an efficient real-time hardware implementation in FPGA, the nonlinear differential equations of the mechanoreceptor spiking model are simulated in MATLAB. Then, the designed digital circuit which provides a multi-module parallel architecture for mechanoreceptor model is executed in VIVADO simulation environment. Using several simulations in different conditions, it is demonstrated that the digital circuit mimic the dynamical behavior of the mechanoreceptor spiking model simulated in MATLAB and the results are in good agreements both quantitatively and qualitatively. Finally, we provide an experimental set up to investigate the performance of the artificial SA-I and FA-I mechanoreceptors in hardware. We present a quantitative justification of the computational accuracy and also show the physical execution results.

The rest of the paper is organized as follows: in section Materials and Methods, the biological concepts and mathematical models of the mechanoreceptor cells are explained. The proposed digital circuit is also described in this section. In section Results of Software and Hardware Simulations, the results of simulations are discussed. Then, section Hardware Implementation, will describe an experimental set up to assess the real-time performance of the digital mechanoreceptor in physical hardware realization. Finally, section Conclusion concludes the paper.

## Materials and methods

In this section, first we explain spiking models of mechanoreceptors and then present digital circuit for hardware realization.

### Mechanoreceptor spiking model

Touch sensing is essential for survival and development of multicellular organisms. Forces that affect the skin are encoded by specialized mechanosensory cells. These touch receptors in our fingertips which are selective, sensitive and fast, allow us for fine tactile sensing and manipulating objects (Kim et al., 2011; Weber et al., 2013). Depending on this skill, we are able to do numerous tasks ranging from ordinary (typing an e-mail) to superior (playing a Mozart concert).

**Figure 1:**
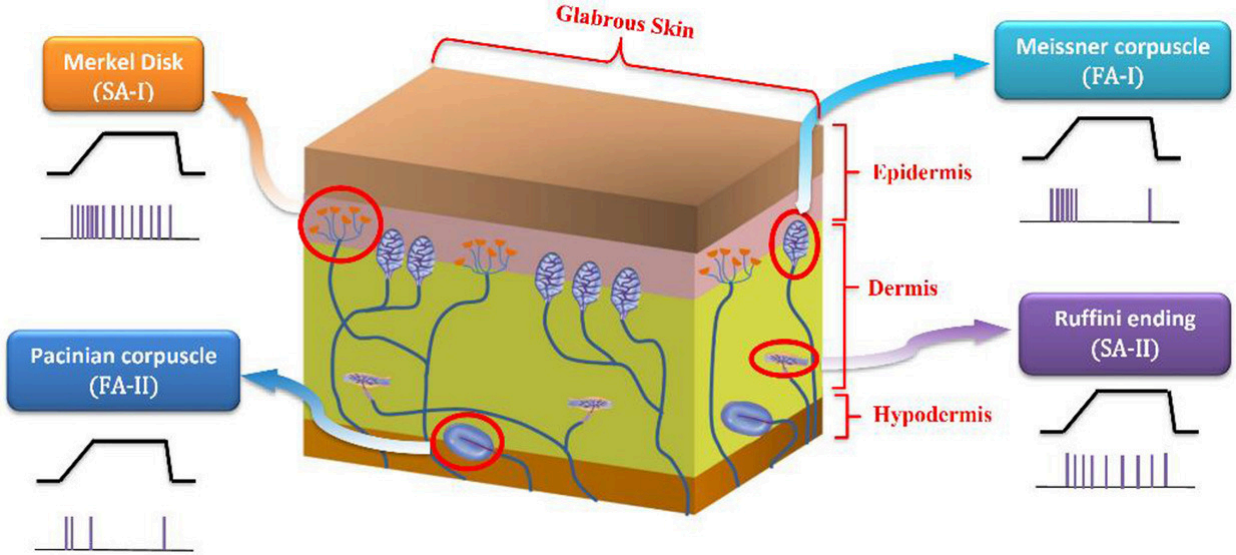
A cross section of the glabrous skin which shows individual type of mechanoreceptors. The obtained spike trains in response to a specific stimulus are also shown.

Slow adapting receptors (SA-I and SA-II) are light-touch receptors which respond to static pressures and thus fire throughout sustained mechanical stimuli. Fast adapting receptors (FA-I and FA-II) are the other touch receptors that respond at the onset and offset of the mechanical stimuli. They respond to vibrations and dynamic forces (derivative of force with respect to time). SA-I receptors are located near the skin surface and respond to its indentations with high sensitivity. SA-II receptors are located deeper inside the skin and are mainly responsible for measuring skin stretch; thus, they are important for proprioception. Both SA-I and FA-I fibers have small receptive field while SA-II and FA-II fibers respond to stimulation of large swaths of skin (Lesniak et al., 2014; Saal and Bensmaia, 2015). Figure 1 shows a cross section of the glabrous skin.

Primary afferent signals are handled by neurons in the cuneate nucleus (CN) of the brainstem, the brain's first level of tactile processing, which organizes the important synaptic relay along the somatosensory pathway from the fingertip to the CNS. The functional link between the first and the second order neurons (mechanoreceptors and cuneate cells, respectively) has not been completely explored, and computational and experimental findings on how information is processed along this pathway are still required (Weber et al., 2013; Saal and Bensmaia, 2014).

To mimic this biological representation, different models have been proposed (Friedl et al., 2016). The mechanoreceptor model by Kim et al. (2013) has been shown to accurately reproduce the spike trains of FA-I and SA-I type cells on a variety of stimuli. Considering this model and the other related models (Kim et al., 2012; Rongala et al., 2015; Friedl et al., 2016; Yi et al., 2017), we take the sensor output *f(t)*, and its derivative ḟ(t), and separate each of them into positive and negative rectified parts producing four signals. The rectified signals are then weighted and summed to make the current [I(t)] to an Izhikevich neuron model. In this way, the sensor-detected force, *f(t)* and change in the detected force, ḟ(t), in N/ms, are linearly converted into current, I(t), in mA. It should be pointed out that we used the previously published Izhikevich model which was shown that is able to reproduce both spiking and bursting responses of the two general types of mechanoreceptors considered in this research (Oddo et al., 2011, 2016, 2017; Rongala et al., 2015; Yi et al., 2017). Although some papers used LIF model (Kim et al., 2009; Bologna et al., 2011, 2013) which is simpler than Izhikevich model, however, the LIF models are not able to accurately reproduce mechanoreceptor diverse responses obtained in experimental observations.

In this design, the Izhikevich neuron model is used due to its capability to exhibit adaptation, which is a key feature of mechanoreceptors, and also to reproduce the dynamic characteristics of the both spiking and bursting responses. The dynamics of the membrane potential, *v*, of the SA-I mechanoreceptors are as follows: (Ranjbar and Amiri, 2016);

(1) $$\begin{array}{l} \frac{dv(t)}{dt}=0.04V{\left(t\right)}^{2}+5V\left(t\right)+140-u\left(t\right)+\frac{K1}{C_{m}}I(t) \end{array}$$

(2) $$\begin{array}{l} \frac{du(t)}{dt}=a(bv\left(t\right)-u\left(t\right)) \end{array}$$

and we have the auxiliary equation as follow:

(3) $$\begin{array}{l} \text{If }v\geq 30mv\{\begin{array}{c} v\leftarrow c\\ u\leftarrow u+d \end{array} \end{array}$$

where *a, b, c, d* are neuron parameters and their values are listed in the Table 1. *u* is the membrane recovery variable and *I* is the input current. The value of the parameters *a* and *b* can be varied to reproduce different kinds of adaptation: *a* defines the characteristic time of recovery variable, *b* defines the sensitivity of recovery variable. In case that the membrane potential reached the threshold value (*V*_*th*_ = 30 mV), one spike was generated and the membrane voltage and the recovery variable are reset according to (3). Parameters *c* and *d* contribute as well in defining the adaptation properties of the neuron. The values of the parameters *a, b, c*, and *d* are chosen to obtain regular spiking and bursting dynamics (Izhikevich, 2003), which is the case of human finger mechanoreceptors. Computations are performed in MATLAB with a time step, dt = 0.01 ms. Similarly, following model matches the spiking activity of FA-I mechanoreceptor cells as discussed in detail in Rongala et al. (2015) and Oddo et al. (2017) and Yi et al. (2017).

(4) $$\begin{array}{l} \frac{dv(t)}{dt}=0.04V{\left(t\right)}^{2}+5V\left(t\right)+140-u\left(t\right)+\frac{K2}{C_{m}}\frac{dI(t)}{dt} \end{array}$$

(5) $$\begin{array}{l} \frac{du(t)}{dt}=a(bv\left(t\right)-u\left(t\right)) \end{array}$$

(6) $$\begin{array}{l} \text{If }v\geq 30mv\{\begin{array}{c} v\leftarrow c\\ u\leftarrow u+d \end{array} \end{array}$$

Indeed, these spiking models of the mechanoreceptor cells have shown promise as computationally efficient models to reproduce a wide range of neural responses to stimuli (Kim et al., 2012; Rongala et al., 2015; Friedl et al., 2016; Yi et al., 2017). In sum, two kind of mechanoreceptors namely, SA-I and FA-I models which are described by the Equations (1)–(6), are used to encoded the input force.

**Table 1:** Parameter values of spiking model of SA-I and FA-I mechanoreceptors used in the simulations.

**Parameter**	**Spiking**	**Bursting**
a	0.02	0.02
b	0.2	0.2
c	−65	−50
d	6	1.5
*V*_*th*_	30 mV	30 mV
*C*_*m*_	1	1

### Digital neuromorphic mechanoreceptor

In this section, we present a digital mechanoreceptor circuit with a new architecture based on the mechanoreceptor spiking model. This digital framework might be implemented on low-cost and commonly available hardware platforms such as FPGAs. Computation methods used in Von Neumann PCs or SIMD processing units such as GPUs or DSPs significantly differ from classic methods used for FPGA (Yang et al., 2016). FPGA, not only implement a real-time platform with the flexibility of programmable logic but also its ability in parallel, high-speed computation, make it as a good choice for designing neuromorphic systems (Nazari et al., 2015c). Indeed, FPGAs can significantly improve the speed of signal processing compared with the software-based methods. In recent years, implementation of digital neuronal networks on FPGAs have attracted considerable attention and several successful cases have been reported in literature (Sabarad et al., 2012; Nazari et al., 2014b).

The digital circuit for the Merkel (SA-I) mechanoreceptor model is obtained first by discretizing its spiking model, namely Equations (1)–(3) using Euler method. The discrete equations are as follows with *h* = *0.01 ms*:

(7) $$\begin{array}{l} v\left[n+1\right]=v\left[n\right]+h*(0.04*v\left[n\right]*v\left[n\right]+5*v\left[n\right]\\ \;+140-u\left[n\right]+\frac{K_{1}}{C_{m}}I\left[n\right]) \end{array}$$

(8) $$\begin{array}{l} u\left[n+1\right]=h*\left(0.02*\left(0.2*v[n]-u[n]\right)\right)+u[n] \end{array}$$

Similarly, discretizing the Meissner's Corpuscle (FA-I) mechanoreceptor spiking model yields:

(9) $$\begin{array}{l} v\left[n+1\right]=v\left[n\right]+h*(0.04*v\left[n\right]*v\left[n\right]+5*v\left[n\right]+140\\ \;-u\left[n\right])+\frac{K_{2}}{C_{m}}(I\left[n+1\right]-I\left[n\right]) \end{array}$$

(10) $$\begin{array}{l} u\left[n+1\right]=h*\left(0.02*\left(0.2*v[n]-u[n]\right)\right)+u[n] \end{array}$$

Considering Equations (7)–(10), Figure 3 shows the scheduling diagram for (a) Merkel Cells (SA-I), (b) Meissner's Corpuscle (FA-I). This figure describes the essential steps to produce the membrane potential (*v*) and the recovery variable (*u*) of the mechanoreceptor model in one iteration. This process is done for producing individual output sample using last samples. At each block a memory register is used to store the outputs which will be utilized in the next computing steps. Each state variable is solved in N-bits registers. “*N*” is the register length and is determined by the required precision for implementation. It directly affects the computational time and cost. In this research, we set *N* = 32 to obtain a low-error, low-cost and high speed circuit. Finally, the desired signals are converted to analog signal using a digital-to-analog converter chip. In this work, the MAX5216PMB1 module was used. This digital system based on reproducing mechanoreceptor dynamics, suggests the neuromorphic conversion of input signal (sensor outputs) to spike/burst patterns conveying tactile information as it is observed in natural touch coding.

**Figure 2:**
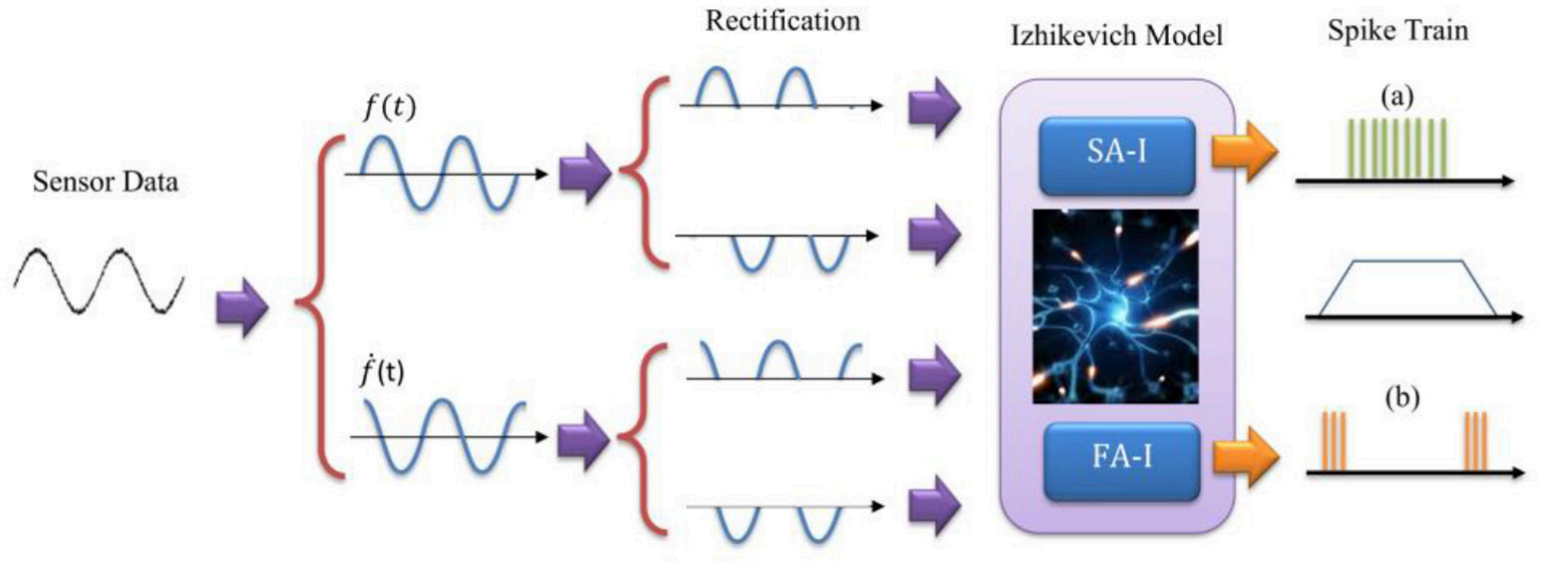
The model for Merkel Cells (SA-I) and Meissner's Corpuscle (FA-I) mechanoreceptor. The FA-I receptor responds with action potentials during stimulus onset and offset. The SA-I receptor is active throughout the period of stimulus contact. The Izhikevich model was used for producing spiking/bursting responses.

**Figure 3:**
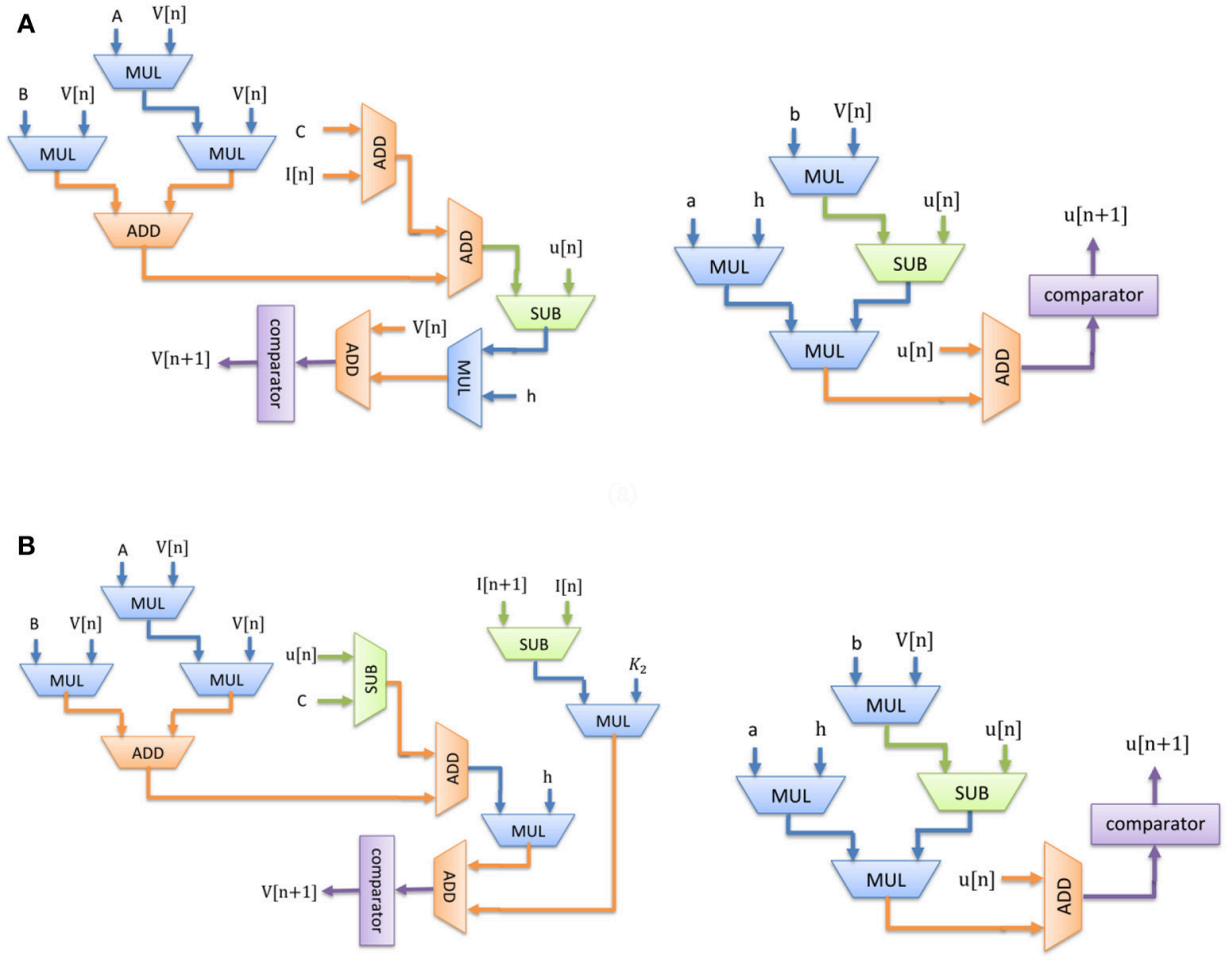
Scheduling diagram for spiking part of the **(A)** Merkel cell (SA-I), **(B)** Meissner's Corpuscle (FA-I). Membrane potential (v dynamic) and the membrane recovery variable (u dynamic).

Considering Figure 3, since there is no high-cost operation to slow critical paths, the reduction in area and increase in maximum operation frequency is expected. Consequently, less hardware resources are required for the proposed digital mechanoreceptor. The digital circuit proposed in this study for matching the dynamic characteristics of the spiking model of mechanoreceptors, can also be extended to different types of *in silico* designs with similar complexity of spiking neuron.

## Results of software and hardware simulations

In this section, the results of software simulations of the mechanoreceptor spiking model in MATLAB and the proposed digital neuromorphic circuit in VIVADO are described. We illustrate how the digital circuit preserves the necessary properties of its spiking counterpart. Both MATLAB and VIVADO simulations were performed using the same dt = 0.01 ms.

**Figure 4:**
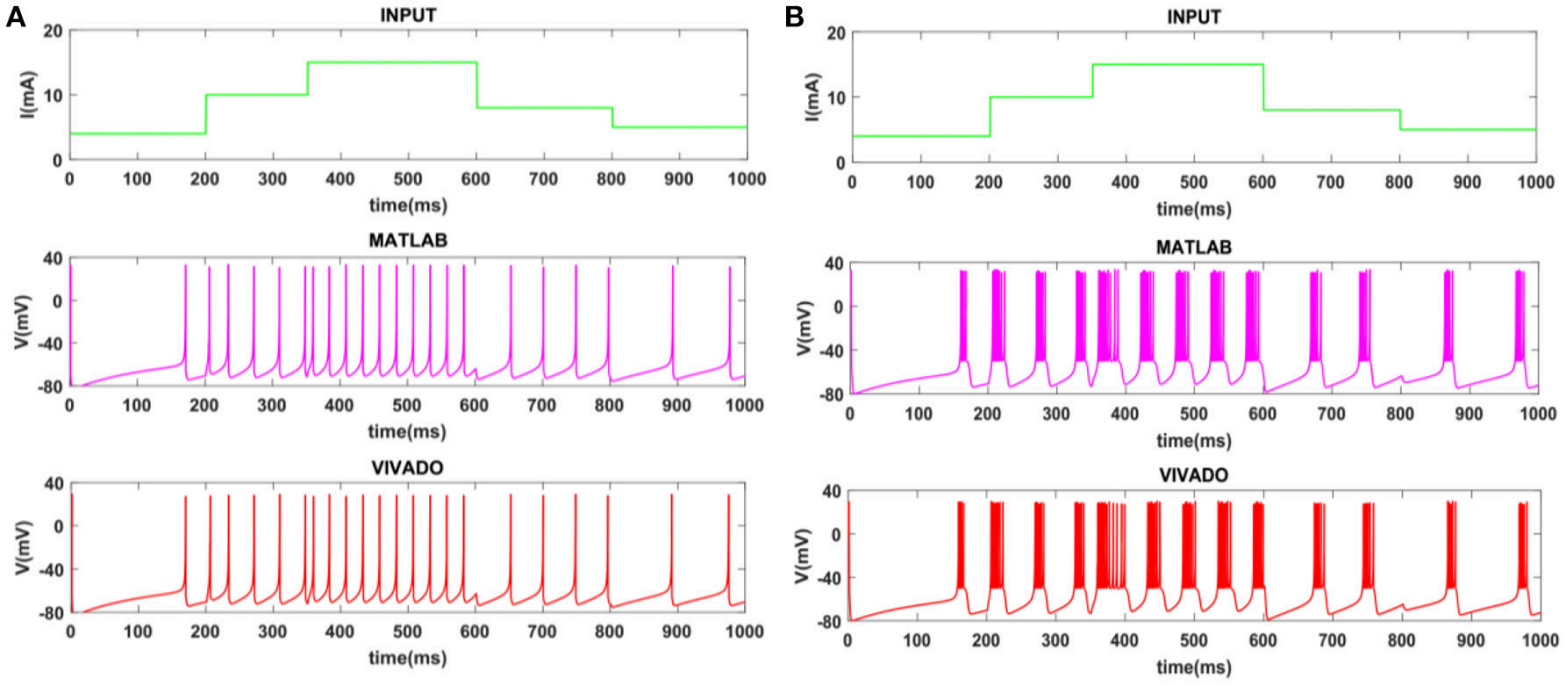
The time response of the Merkel Cells (SA-I) mechanoreceptor in mV. **(A)** Spiking and **(B)** bursting response. In these simulations, the first panels show the input signal, the second panels display the MATLAB simulation of the spiking mechanoreceptor model and the third panels illustrate the VIVADO simulation of the proposed digital circuit.

**Figure 5:**
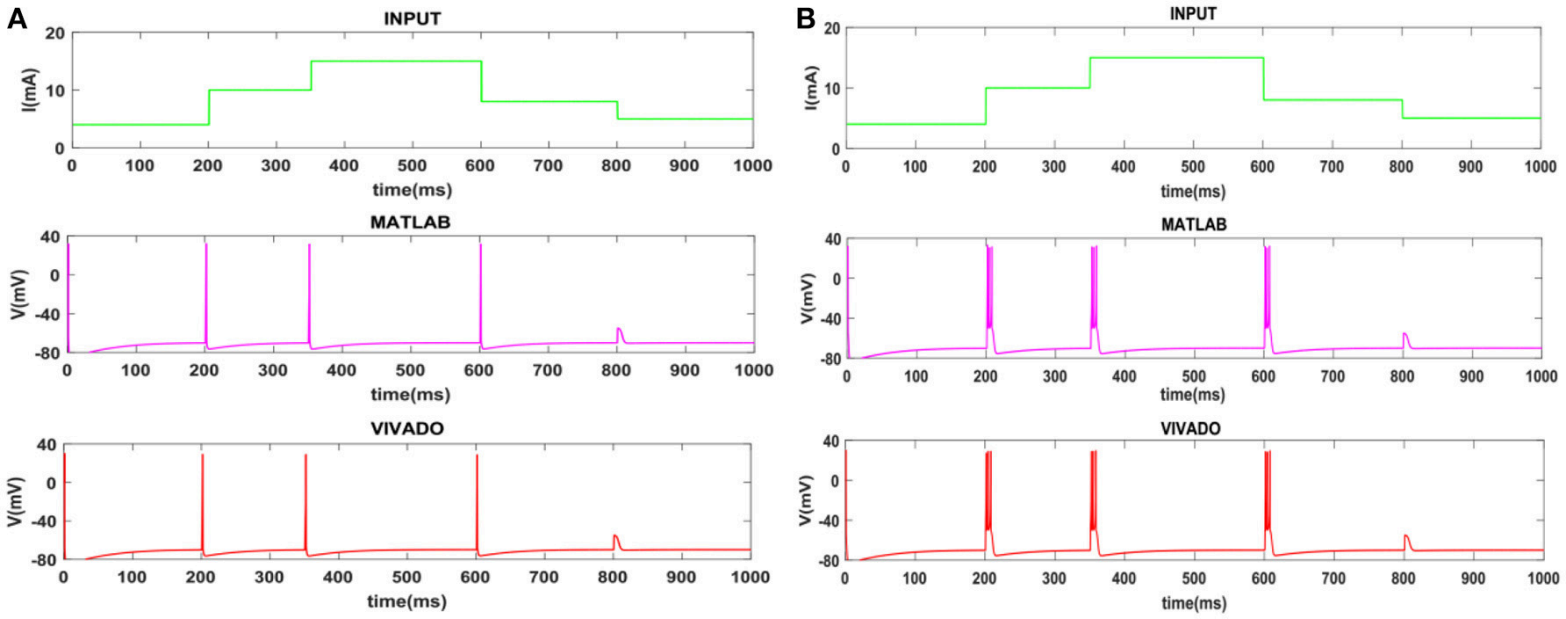
The time response of the Meissner's Corpuscle (FA-I) mechanoreceptor in mV. **(A)** Spiking and **(B)** bursting responses. In these simulations, the first panels show the input signal, the second panels display the MATLAB simulation of the spiking mechanoreceptor model and the third panels illustrate the VIVADO simulation of the proposed digital circuit.

To show the flexibility of the designed circuit and to compare its capabilities and behavior with those of mechanoreceptor spiking model, several simulations are done. Figures 4, 5 show the spiking and bursting responses for Merkel Cells (SA-I) and Meissner's Corpuscle (FA-I), respectively. In these figures, the first panels show the staircase pulse as the input signal, the second panels display the MATLAB simulations of the spiking mechanoreceptor model and the third panels illustrate the VIVADO simulation of the designed digital circuit. According to these results, both spiking and bursting responses could be realized and hence the digital circuit is able to work in both regimes. It should be pointed out that the model parameters for spiking and bursting patterns are shown in Table 1.

Considering Figures 4, 5, the SA-I receptors fire throughout a sustained stimulus and the FA-I receptors respond at the onset and offset of that stimulus. This result is in agreement with the response obtained by the observations reported in Jöntell et al. (2014). In other words, the spiking mechanoreceptor model which is inspired from the biology of human tactile perception, and the proposed a digital mechanoreceptor circuit, produce time responses which functionally are in agreement with spiking activity of mechanoreceptor cells.

**Figure 6:**
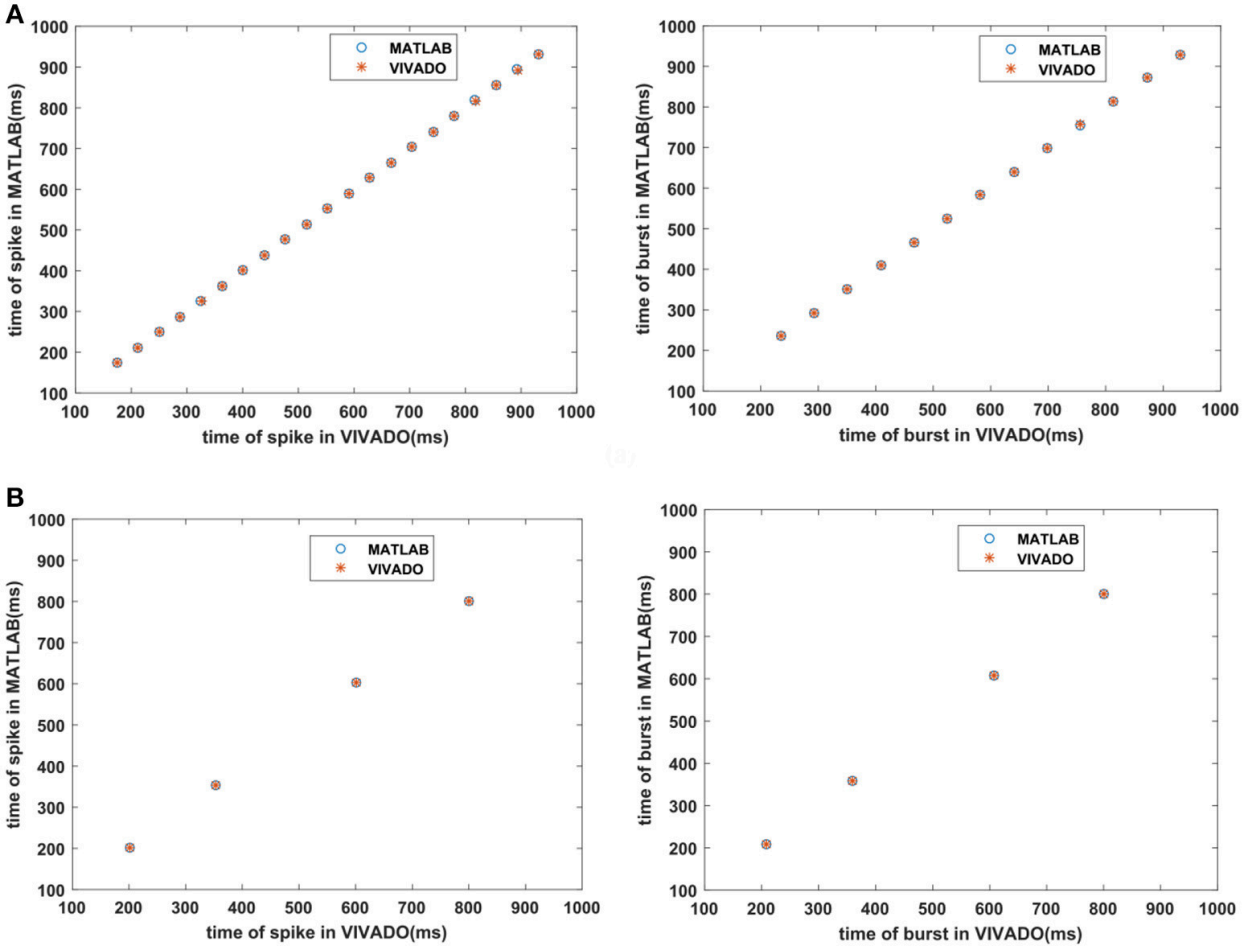
The timing of firing **(A)** the SA-I mechanoreceptor, **(B)** the FA-I mechanoreceptor for spiking/bursting responses obtained by the MATLAB simulations of the SA-I and FA-I mechanoreceptor models and VIVADO simulation of the proposed SA-I and FA-I digital mechanoreceptors. This figure corresponds to Figures 4, 5.

**Figure 7:**
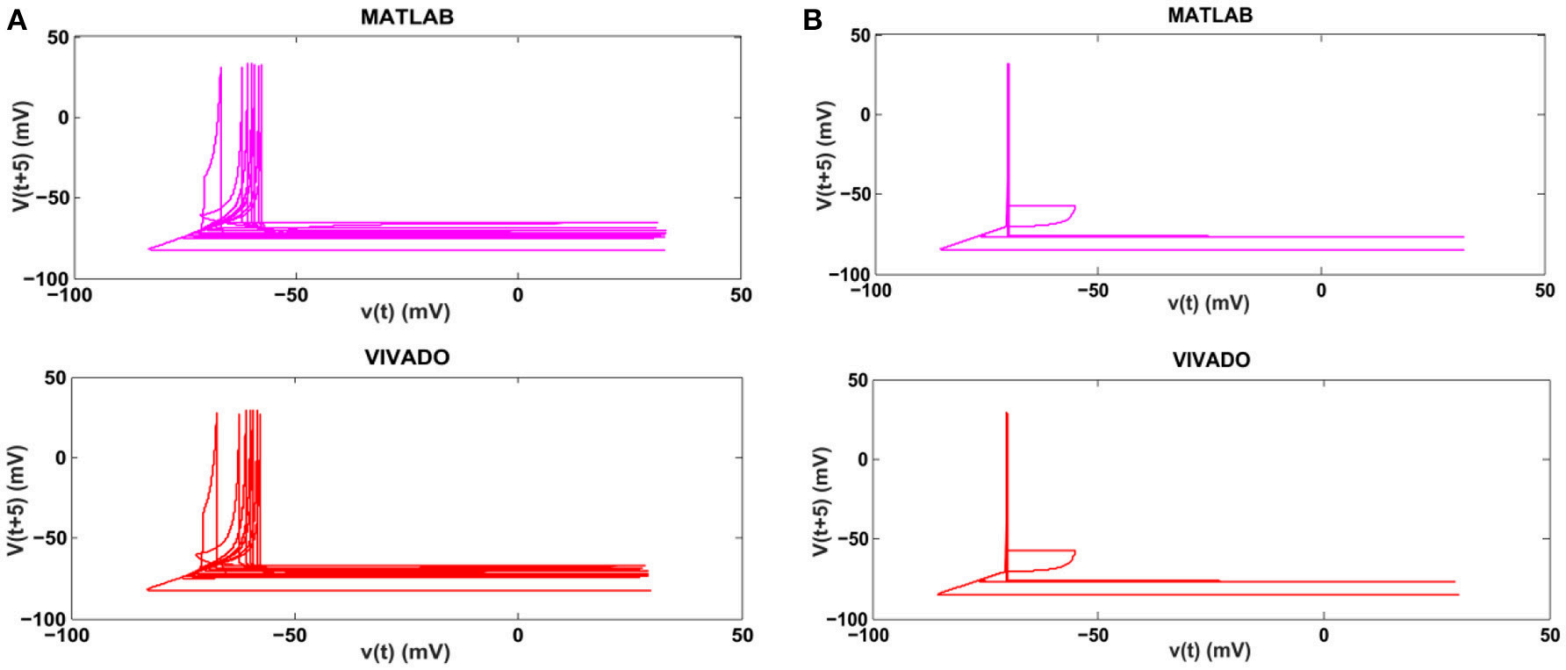
The phase plane of **(A)** the SA-I mechanoreceptor, **(B)** the FA-I mechanoreceptor for spiking responses. In each part, first panel shows the mechanoreceptor model simulated in MATLAB and the second panel displays the mechanoreceptor digital circuit simulated in VIVADO. This figure corresponds to Figures 4, 5A, for spiking mode. It can be seen that the proposed circuit preserves the model dynamics.

**Figure 8:**
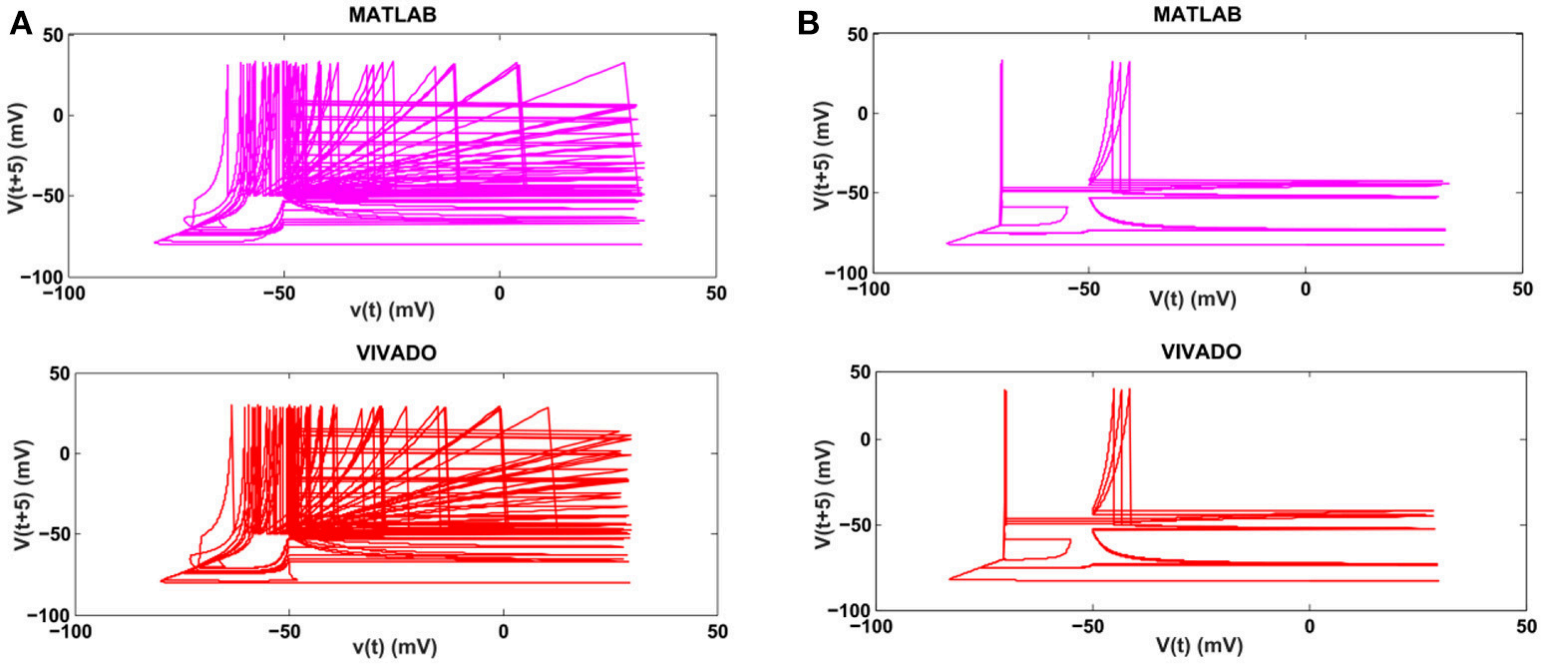
The phase plane of **(A)** the SA-I mechanoreceptor, **(B)** the FA-I mechanoreceptor for bursting response. In each part, first panel shows the mechanoreceptor model simulated in MATLAB and the second panel displays the mechanoreceptor digital circuit simulated in VIVADO. This figure corresponds to Figures 4, 5B, for bursting mode. It can be seen that the proposed circuit preserves the model dynamics.

Next, we continue our simulations and compare the result of MATLAB and VIVADO simulations. To this end, we compute the mean and variance of Inter-spike interval (ISI) and Inter-burst interval (IBI) of the results shown in Figures 4, 5, which are reported in Table 2. Indeed, the responses of any neuron can be characterized by the spike timing and these spiking responses carry information (Jöntell et al., 2014; Saal and Bensmaia, 2014). Consequently, ISIs and IBIs are important factors to be considered and compared to validate the reliability of responses obtained by the proposed digital circuit. Table 2 shows the mean and variance values of ISIs and IBIs obtained by the MATLAB simulations of the mechanoreceptor model and VIVADO simulation of the proposed digital mechanoreceptor. In this way, Figure 6 shows the timing of firing a spike or burst obtained by the MATLAB and VIVADO simulations of the mechanoreceptor model and its digital circuit, respectively which illustrates a good agreement. Given Table 2 and Figure 6, we expect that the errors caused by the approximation of the discrete equations are small and thus the spike timing (a fundamental component in brain information processing) is matched. Finally, the performance of the proposed digital circuit from dynamical point of view is investigated. Figures 7, 8 show the phase plane, *v(t*+*5*) vs. *v(t)*, of the mechanoreceptor models simulated in MATLAB and the digital mechanoreceptors for spiking and bursting responses, respectively. As can be observed, although there are also some quantitative differences, the overall shape and features of the trajectories are similar, qualitatively. Regarding the obtained results, one can conclude that the designed circuit has maintained the dynamical characteristics of the original system.

**Table 2:** The average and variance values of ISI and IBI for two types of mechanoreceptors using VIVADO and MATLAB simulations.

		**ISI**		**IBI**	
		**Average**	**Variance**	**Average**	**Variance**
SA-I	MATLAB	37.8550	0.0025	57.8538	0.0025
	VIVADO	37.8400	0.0024	57.8538	0.0025
FA-I	MATLAB	199.7000	1633.5800	197.5667	1620.0156
	VIVADO	199.7000	1633.5800	197.6000	1623.4400

Considering Figures 4–8, the proposed digital circuit properly demonstrates similar time domain and dynamical behaviors of its computational model without any serious limitation. This operational circuit can effectively be executed in an FPGA device (in the next section). It is capable of realizing both spiking and bursting responses with a few number of multipliers to decrease the hardware resource requirement. This highlights that a large number of digital mechanoreceptor can be realized on an FPGA in real time.

## Hardware implementation

**Figure 9:**
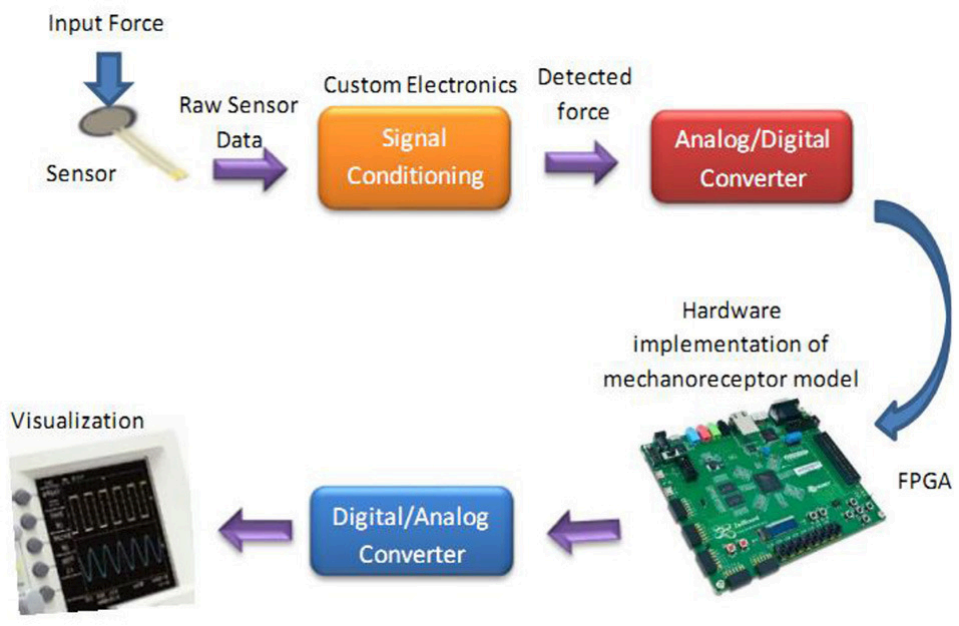
The diagram for hardware testing of the mechanoreceptor digital circuit. In this case, the digital mechanoreceptor is implemented on the ZedBoard and the obtained signals after converting to analog signal will be displayed on oscilloscope. A 10-bit ADC was used for analog to digital conversion. However, a 16-bit DAC was used to convert the digital outputs of the ZedBoard to analog signals to be shown on the Oscilloscope.

The whole diagram for hardware testing of the proposed digital mechanoreceptor is shown in Figure 9. This platform encodes the force recorded from sensor into the spiking activity of a mechanoreceptor digital circuit. Indeed, detected force at the sensor is converted into current, which in turn produces a train of action potentials. This is analogous to how stress and/or strain applied at a mechanoreceptor end organ is transformed into current across its membrane.

To verify the validity of the proposed digital design for the mechanoreceptor model, it has been executed on the ZedBoard development kit. The expandability features of this platform make it possible for proof-of-concept development and rapid prototyping. The key objective is to explore the feasibility of FPGA implementation of the circuit, specifically targeted to benefit from reconfigurable hardware blocks and parallel processing. The first component in Figure 9 is the force sensor. Force-sensitive resistor (FSR) is commercially available and has relatively easily integration with peripheral hardware and software. It designed for measuring the presence and relative magnitude of localized physical pressure. FSR separated by two layers, as the pressure increases, these points will touch the active semiconductor elements, so that the resistance becomes small. In other words, it can be seen as a resistor to change its resistance value by the size of the pressure of the resistance (in ohms, Ω) depends on how much repression. FSR responds to normal force within a range of 0.2–20 N over its thin and circular pressure sensitive area. The voltage passed through the sensor is first amplified and then filtered before being fed to a 10-bit ADC (analog-to-digital converter), which collects data at a 200 kHz sampling rate. Supporting software (VIVADO) was developed to read the digital signal from ADC to be prepared for real-time execution of the digital mechanoreceptor implemented on the ZedBoard.

Force detected at the sensor, *f(t)*, is transformed into current (Figure 2) to be injected as the input current to the digital mechanoreceptor. Following the procedure mentioned in (Rongala et al., 2015), a broad range of values for gain factors (*K*_1_ and *K*_2_) have been tested. High gain values induced a strong firing rate independent from the stimulus and results in a less informative temporal structure of spikes. However, low gain factors lead to low firing rate and consequently to a long latency in spike responses (Oddo et al., 2017). After proper tradeoff, we achieved *K*_1_ = 0.75 and *K*_2_ = 3. Next, the input current is transformed into spike/burst trains using digital mechanoreceptor implemented on the ZedBoard.

**Figure 10:**
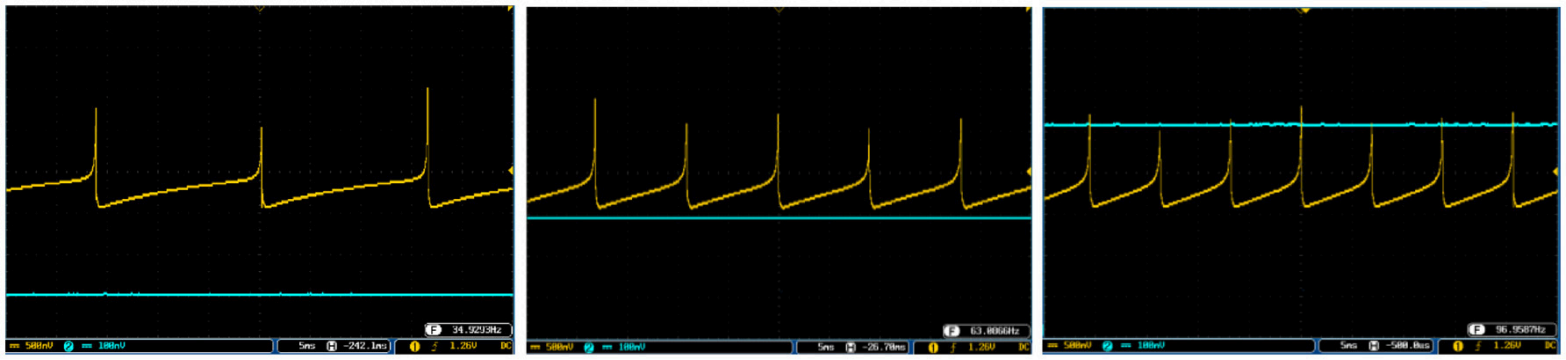
The spiking response of the digital SA-I mechanoreceptor (yellow color) executed on the ZedBoard. Signals are physically produced and observed on the oscilloscope. The SA-I mechanoreceptor remains active during the period of stimulus contact. The filtered input of the A/D is shown in blue. The volt division for the output (input) channel was set on 500 mV (100mV).

**Figure 11:**
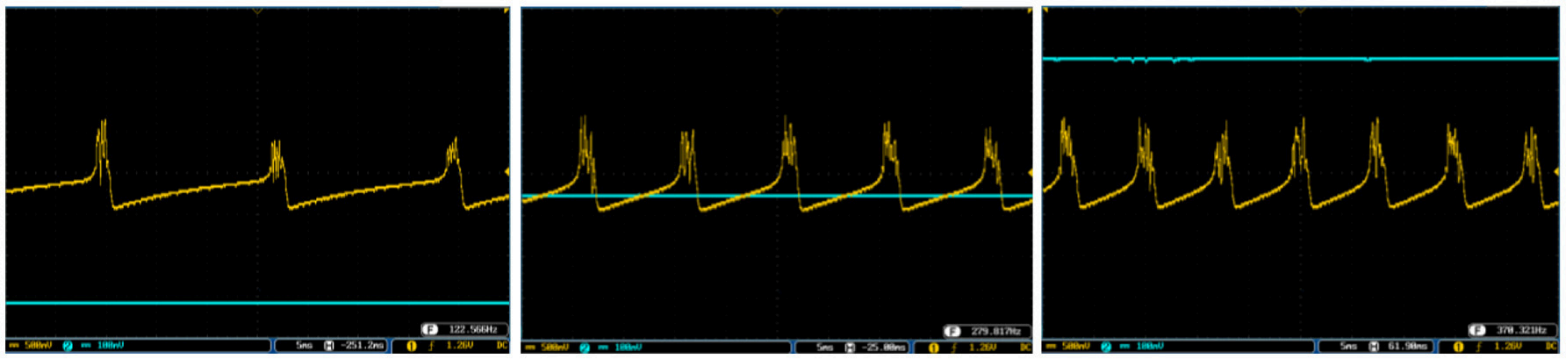
The bursting response of the digital SA-I mechanoreceptor (yellow color) executed on the ZedBoard. Signals are physically produced and observed on the oscilloscope. The filtered input of the A/D is shown in blue. The volt division for the output (input) channel was set on 500 mV (100mV).

**Figure 12:**
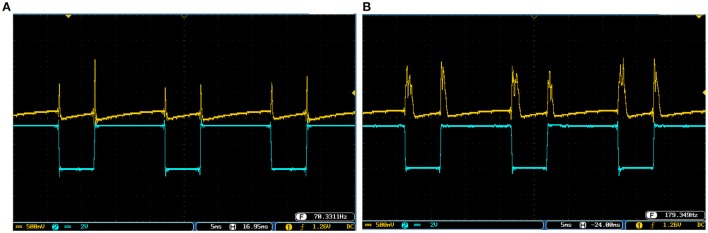
The output of the digital FA-I mechanoreceptor (yellow color) implemented on the ZedBoard for **(A)** spiking and **(B)** bursting responses. Signals are physically produced and observed on the oscilloscope. The FA-I mechanoreceptor responds with bursting / spiking patterns during stimulus onset and offset (blue color). The volt division for the output (input) channel was set on 500 mV (2V).

Figures 10, 11 show oscilloscope photographs of the digital realization of the SA-I mechanoreceptor for spiking and bursting responses, respectively. In these figures, the output of the FPGA board was shown in yellow color (membrane voltage of mechanoreceptor) and the filtered input of the A/D is shown in blue. As it is observed, as the amplitude of the detected force increases, the frequency of the spiking/bursting patterns is also increased. This approach makes possible to decode stimuli while the tactile data stream is collected. This in fact is in agreement with experimental observations in which different stimulation patterns evoked different total number of spikes (Weber et al., 2013; Jöntell et al., 2014; Saal and Bensmaia, 2014). The results obtained for digital realization of the FA-I mechanoreceptor are shown in Figure 12. The device utilization for realization of both SA-I/FA-I digital circuit is summarized in Table 3.

**Table 3:** Device utilization summary of the ZedBoard.

	**Used in SA-I**	**Used in FA-I**	**Available**
Slice LUTs	975	1131	53,200
Slice Registers	65	97	1,06,400
Slice	268	309	13,300
LUT Flip Flop Pairs	60	60	53,200
DSP48	16	18	220
Bonded IOB	12	12	200

**Figure 13:**
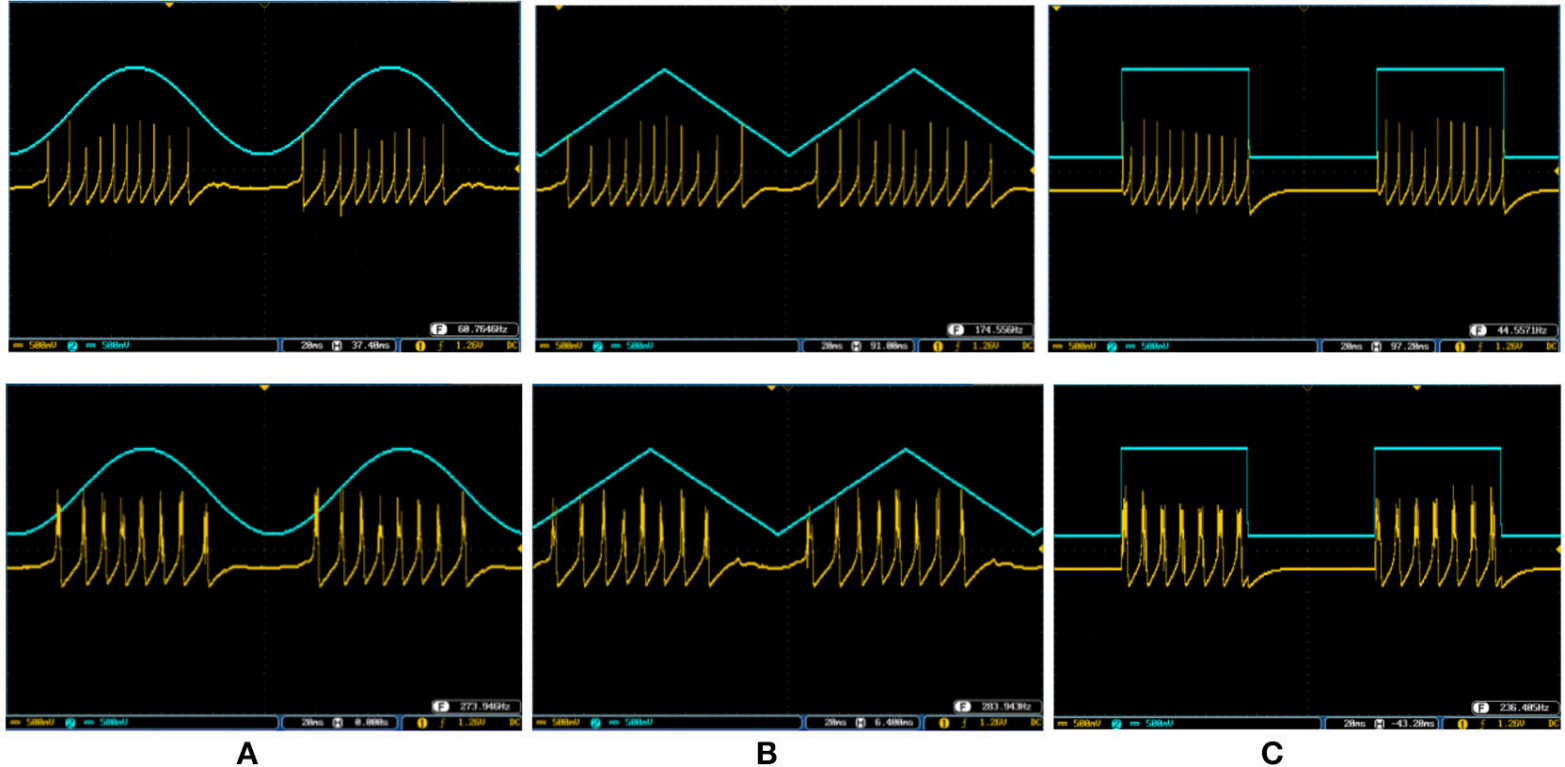
The spiking and bursting responses of the digital Merkel (yellow color) executed on the ZedBoard when it receives different input signals. The input is shown in blue (1mA, p-p). **(A)** Sinusoidal, **(B)** triangular, and **(C)** pulsatile input. The volt division for both output and input channels was set on 500 mV.

**Figure 14:**
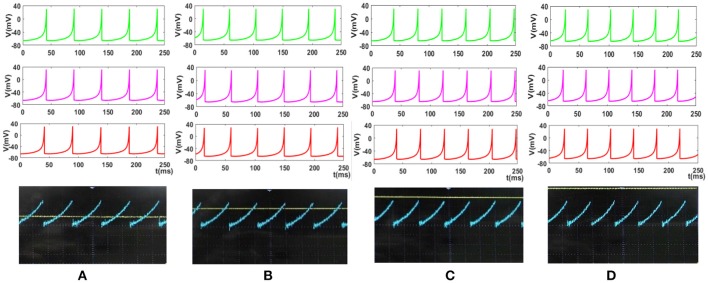
The response of the Merkel spiking model. In each part, the first panel (green) shows the response of the differential Equations (1) and (2) solved by Runge–Kutta method, RK4, the second panel (pink) is the response of the discrete Equations (7) and (8), the third panel (red) illustrates the VIVADO simulation of the digital circuit and the last panel is the response of the digital mechanoreceptor (blue color) executed on the ZedBoard. The last panel also displays the input (yellow color) which its amplitude is for **(A)** 2.4, **(B)** 2.6, **(C)** 2.8, **(D)** 3 mA. The Time division was set on 25 ms.

To cover more input signals in addition to the step signal, different inputs such as sinusoidal, triangular and pulsatile are also applied to FPGA when it is running mechanoreceptor digital circuits. Figure 13 shows the output of digital Merkel (yellow color) while input signal is in blue color. Depending on the amplitude and frequency of the input, digital mechanoreceptor sends a train of spikes/bursts to the output pin of the ZedBoard to be shown on the oscilloscope (after analog conversion). To provide quantitative analysis, the physical outputs of the digital mechanoreceptors (ZedBoard) are compared with MATLAB simulation of continuous (Equations 1, 2 solved by Runge–Kutta method, RK4) and discrete (Equations 7, 8) spiking models and VIVADO simulation of the digital circuit (Figure 3) for the same input. An input signal with four different amplitudes is used for performance comparison. Figure 14 illustrates the obtained responses which are completely matched for a specific input. To do a comparison of the firing patterns produced by the digital realization to those of the computational models, the ISI values are computed and reported in Table 4. As discussed previously, the ISIs are important factors to be compared for reliability validation of the responses. The very low relative error (Table 4, last column) between the ISI values obtained by the MATLAB/VIVADO simulations and digital realization on an FPGA, indicates an acceptable performance and thus the proposed digital circuit is faithful.

**Table 4:** The values of ISI (in *ms*) for spiking responses calculated using MATLAB and VIVADO simulations and FPGA implementation corresponds to different parts of Figure 14.

** 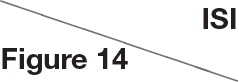 **	**MATLAB**		**VIVADO simulation of Figure 3**	**Physical implementation on the ZedBoard**	**Relative Error**
	**Continuous Diff. Equations (1) and (2) solved by RK4**	**Discrete Diff. Equations (7) and (8)**			
**A**	48.6	48.6	48.7	48.5	0.002
**B**	44.5	44.7	44.9	45	0.011
**C**	41.2	41.3	41.3	41.5	0.007
**D**	38.5	38.4	38.5	39	0.012

*Relative error is computed by considering the output of the ZedBoard (5th column) as the measured value and obtained value by RK4 (2nd column) as the actual value and then use RE = |actual value−measured value|/actual value*.

Although this analysis does not support the validity of the model from biological point of view, it shows that digital circuit executed on the FPGA properly follows the spiking model, which is a necessary step for moving forward and further analysis. Considering Figures 10–14, the digital circuit maintains the essential properties of its computational counterpart in different conditions. Regarding the main criteria from the hardware viewpoint such as scaling up the circuit, decreasing the digital realization cost while obtaining results similar to the mechanoreceptor computational model, the digital circuit produces satisfactory responses. Finally, this neuromorphic approach can offer the possibility to mimic a sense of touch with flexible design features to evaluate related effects. This also supports the design of new architectures for artificial tactile sensory systems for rehabilitation applications.

## Conclusion

Considering performance, power and time constraints, recent improvements in FPGA technology support flexibility required for algorithm exploration. By discovering the basic mechanisms found in the neuroscience and transforming them to hardware realization, it is possible to advance current technologies. These neuro-inspired novel technologies have several real-world applications including adding sensory capabilities to provide information about body positioning (proprioception) and grip forces (Jöntell et al., 2014; Raspopovic et al., 2014; Oddo et al., 2016).

The present research opens a new window to analyze mechanoreceptors in hardware. To overcome the problems of analog fabrication, in this research, a digital execution was used. We proposed a digital neuromorphic circuit both in software simulations and hardware realization. It was shown that the system reproduced spike/burst patterns and was mainly oriented for applications requiring efficient and low-power hardware systems. In this way, the proposed circuit enabled us to design hardware architecture for running on an FPGA. The compartmentalized structure of the digital circuit and the ability to control mechanoreceptor parameters facilitated to add supplementary mechanisms without extensive circuit redesign. This helped for easy scalability of the model to include a greater number of mechanoreceptors on an FPGA. This engineering approach is a new method for fabricating sensory systems which artificially replicates the firing activities of the SA-I and FA-I afferents. It should be pointed out that the proposed digital mechanoreceptor has minimal level of biological plausibility in the sense that for the digital Merkel receptor, firing rate increases with higher forces and for the digital Meissner receptor, firing rate changes based on the rate of force changes. Nevertheless, in this digital realization the structure of the mechanoreceptors was ignored and the input/output properties were considered. Furthermore, parameter sensitivity analysis and comparison of the results of the digital realization with biological data should be investigated in future development of this approach.

Future works will be conducted to include the other mechanoreceptor models. Moreover, by implementing a large population of digital mechanoreceptor, the development of new generation of prosthetic hands to reestablish sensory feedback for people with skin damage or amputations can also be possible. The obtained spike/burst trains from digital mechanoreceptors may be passed to a brainstem spiking model (which also can be implemented in hardware) for further processing. This will make a neuromorphic sensory system that will be utilized on a mobile robot to do various real-world tasks such as texture discrimination and object recognition.

## Author contributions

NS-N, MA, EF and CL did conception and design, analysis and interpretation of data, drafting and revising the article. NS-N and MA performed the experiments and acquired the data.

### Conflict of interest statement

The authors declare that the research was conducted in the absence of any commercial or financial relationships that could be construed as a potential conflict of interest.
